# The Impact of Generativity on Maintaining Higher-Level Functional Capacity of Older Adults: A Longitudinal Study in Japan

**DOI:** 10.3390/ijerph20116015

**Published:** 2023-05-31

**Authors:** Kumiko Nonaka, Hiroshi Murayama, Yoh Murayama, Sachiko Murayama, Masataka Kuraoka, Yuta Nemoto, Erika Kobayashi, Yoshinori Fujiwara

**Affiliations:** Tokyo Metropolitan Institute for Geriatrics and Gerontology, 35-2 Sakae-cho, Itabashi, Tokyo 173-0015, Japan

**Keywords:** generativity, higher-level functional capacity, older adults, longitudinal study

## Abstract

Generativity is defined as an individual’s concern for and actions dedicated toward the well-being of others, especially youth and subsequent generations. It is a key stage of psychological development from midlife to older age and can be a guiding concept for promoting engagement of older adults in productive and contributive activities, which benefit their well-being. This study examined the longitudinal association between generativity and higher-level functional capacity (HLFC) decline in older Japanese adults. The two-year longitudinal data of 879 older adults aged 65–84 years were analyzed. Participants’ HLFC and generativity were assessed using the Tokyo Metropolitan Institute of Gerontology Index of Competence and the Revised Japanese version of the Generativity Scale, respectively. The binary logistic regression analysis results showed that a higher generativity score was negatively associated with HLFC decline, indicating that generativity effectively prevents HLFC decline over 2 years. On adding the interaction term between generativity and sex to examine whether the protective effect of generativity differed by sex, we found that generativity was especially effective in protecting the HLFC decline in men with higher generativity. The study results highlight the importance of promoting engagement of older adults in generative activities to maintain their HLFC.

## 1. Introduction

Life expectancy at birth for Japanese individuals has been increasing; as of 2021, it is 87.57 years for women and 81.47 years for men [1]. Due to this increase in longevity, there is a need to ensure their well-being. Japan’s public health policy encourages older adults to engage in productive and contributive activities by capitalizing on their skills and experiences in their community (e.g., volunteering and providing support for others), which would benefit older adults’ well-being whilst strengthening the community’s social capital [2].

Generativity can be key to motivating older adults to engage in contributive acts. The concept of generativity, which is derived from Erikson’s theory of developmental stages [3], proposes eight stages of psychological development across the lifespan. Each stage with an opposing outcome relates to previous and subsequent stages. On successfully completing a certain stage, individuals strengthen their sense of self and increase their competences to deal with life challenges that they will face in the subsequent stages [3,4,5,6,7]. “Generativity vs. stagnation” is the seventh stage that occurs between midlife and older age. Generativity is defined as an individual’s concern for, attitudes and behaviors dedicated to, the well-being of others, especially youth and subsequent generations [3,4]. Absence of generativity is described as a period of stagnation, where individuals fail to become productive and contributing members of their community [3]. Generativity can be achieved through various altruistic and supportive actions and behaviors that individuals often engage in from midlife to old age. These actions can occur within family and public contexts—such as parenting, looking after grandchildren and dependents, providing emotional support, mentoring younger generations, and engaging in volunteer work—thereby productively contributing to others and the community [3,4,5,6,7,8,9]. These activities involve social participation, frequent interactions with others, and physical and cognitive activities, contributing toward older adults’ well-being [6,9,10]. Therefore, promoting generativity is expected to be beneficial for both older adults and society.

Previous studies have examined the association between generativity and health in older adults. For instance, numerous studies explored the positive relationship between generativity and psychological health, such as life satisfaction, sense of self-worth, feeling of usefulness, cognitive affects, and lower levels of depressive and anxious episodes [8,9,11,12,13,14,15]. A study conducted on older Americans aged 60–75 years [10] demonstrated that individuals with greater perceptions of generativity are less likely to experience an increase in Activities of Daily Living (ADL) disability or die over a 10 year-period.

Although studies have demonstrated the protective effects of generativity on psychological health and ADL disability, to our knowledge, no longitudinal study has focused on the effect of generativity on higher-level functional capacity (HLFC) in older adults. It is important for older adults to maintain HLFC, as it is essential to maintain a socially independent lifestyle in one’s community [16]. Studies have reported that HLFC deterioration predicts a decline in the Basic ADL (BADL) [17], and mortality of older adults with normal BADL [18]. Therefore, our study examined the longitudinal association between generativity and HLFC decline in community-dwelling older Japanese adults using longitudinal data.

Additionally, we examined whether the protective effect of generativity differed by sex. Studies have reported that women demonstrate higher generativity than men [8,19], and that generativity is more predictive of psychological health for older women than for older men, as older women have been socialized to be other-oriented and engage in nurturing roles [8].

## 2. Materials and Methods

### 2.1. Study Participants

This study utilized data from a two-year longitudinal study of adults aged 25–84 years in the Kita Ward of Tokyo and the Tama Ward of Kawasaki City in Japan. The Kita Ward is located in the northern part of central Tokyo, with a total population of 344,155 and an aging rate of 25% as of 2016. The Tama Ward is a typical commuter city in the western suburb of the Tokyo metropolitan area, with a total population of 205,275 and an aging rate of 19% as of 2016. As the initial purpose of this longitudinal study was to assess the effects of community-based interventions on increasing social capital and generativity among residents in the two wards, one district in both the Kita and Tama wards was assigned as the intervention area and the remaining districts as control areas [20]. However, the current study aimed to examine the association between generativity and HLFC decline among older adults and not the effects of the intervention programs. Thus, in this study, we used the subset of the study participants aged 65–84 years at baseline (*n* = 6777). Furthermore, we excluded those participants’ data who were residing in the intervention areas (*n* = 589) of both the wards as they were exposed to the intervention programs to increase generativity.

**Figure 1:**
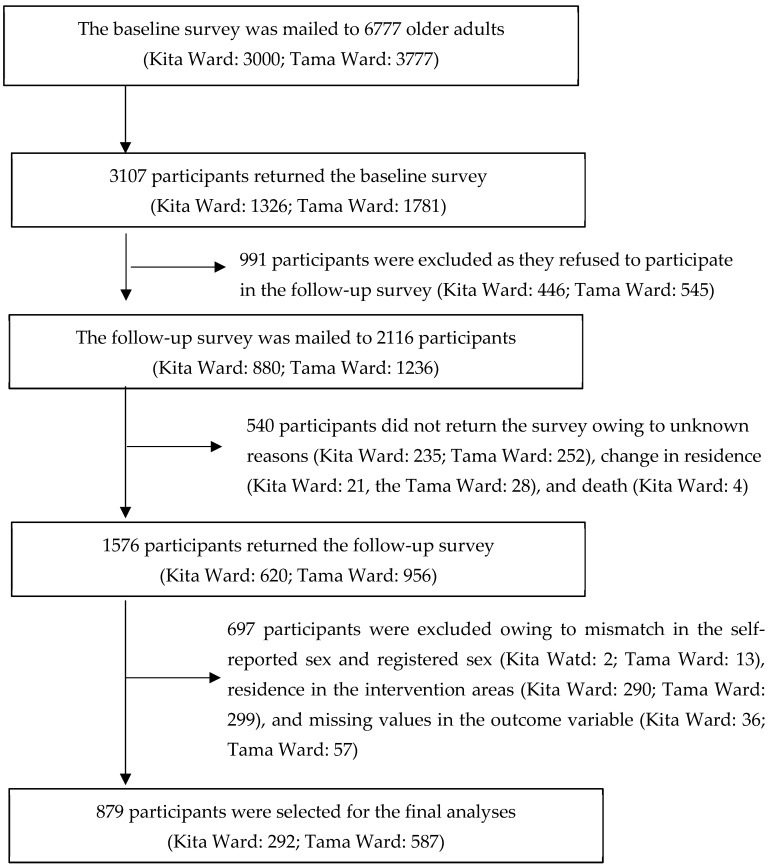
Selection procedure for older adults aged 65–84 years in the baseline longitudinal study and the current study.

Figure 1 illustrates the selection procedure for older adults aged 65–84 years in the baseline longitudinal study and current study. In the baseline study, the inclusion criterion was community-dwelling older adults aged 65–84 years living in either the Kita or Tama wards in 2016; the exclusion criterion was older adults residing in long-term care facilities. For the baseline study, 6777 eligible older adults were randomly selected from the Basic Resident Registration System of the Kita and Tama wards municipalities (Kita Ward: 3000; Tama Ward: 3777), and an invitation letter and self-administered questionnaires were mailed to them in 2016. The invitation letter explained the study’s aim, that is, to examine participants’ attitudes and behaviors toward intergenerational interactions. Participants were requested to complete the questionnaires and participate in the follow-up survey in 2018. Data from 3107 participants (Kita Ward: 1326; Tama Ward: 1781) (response rate: 45.8%) were obtained; those who refused to participate in the follow-up survey were excluded (*n* = 991). Subsequently, the follow-up survey was mailed to 2116 participants (Kita Ward: 880; Tama Ward: 1236). Of the 2116 participants, 540 did not return the questionnaires due to unknown reasons (*n* = 487), change in residence (*n* = 49), and death (*n* = 4). Thus, data from 1576 (74.5%) participants—620 and 956 from the Kita and Tama wards, respectively—were obtained. Furthermore, we excluded data of participants (*n* = 697) whose self-reported sex in questionnaires differed from that registered in the Basic Resident Registration System of the municipalities (*n* = 15), residing in intervention areas (*n* = 589), and with missing values in the outcome variable utilized in the study analyses (*n* = 93). In total, data from 879 participants (Kita Ward: 292; Tama Ward: 587) were analyzed. This study was conducted in accordance with the Declaration of Helsinki and approved by the Ethics Committee of the Tokyo Metropolitan Institute of Gerontology (protocol code 28KEN-1042; date of approval: 1 June 2016).

### 2.2. Measurements

#### 2.2.1. Outcome Variable

Outcomes were defined as the maintenance or decline in HLFC between the baseline and follow-up surveys. HLFC was evaluated using the total score of the Tokyo Metropolitan Institute of Gerontology Index of Competence (TMIG-IC), a 13-item index designed to measure HLFC above ADL, based on Lawton’s model [20]. The response to each item was scored as 0 for “unable to do” and 1 for “able to do” (ranging from 0 to 13), with higher scores representing greater independence. We created a dichotomous variable of change in HLFC as the outcome variable, which comprised two categories: “maintained” and “declined”. The “maintained” category included participants whose TMIG-IC scores at the follow-up survey remained unchanged or improved from the baseline survey. The “declined” category comprised participants whose TMIG-IC scores at the follow-up survey decreased by one or more points from the baseline survey.

#### 2.2.2. Generativity

Generativity was assessed using the Revised Japanese version of the Generativity Scale (JGS-R) [21]. The JGS-R comprises 12 questions, which measure the degree of individuals’ generative concern (e.g., for sharing their own experiences with others) and behaviors (e.g., providing advice to younger generations). The items are rated on a scale ranging from 0 (“strongly disagree” for concern and “not doing at all” for behavior) to 5 (“strongly agree” for concern and “doing quite often” for behavior). The total score ranges from 0 to 60, with higher scores indicating greater generativity [21]. Subsequently, the total generativity scores are divided by 10 to reduce scales; thus, the score ranged from 0 to 6.

#### 2.2.3. Covariates

Covariates were obtained from the baseline survey data, including sex (0 = woman, 1 = man), age, having children (0 = have, 1 = do not have), participants’ residential area (0 = Tama Ward, 1 = Kita Ward), highest educational level, annual household income, self-rated health, and baseline functional health status. For age, participants were divided into two groups (0 = 65–74 years, 1 = 75–84 years). The highest educational level was categorized as “high school graduate or under” and “over high school graduate”, which included vocational school, two-years at college, university, and graduate school. Annual household income consisted of “less than 2 million yen”, “2 million yen to less than 3 million yen”, “3 million yen to less than 5 million yen”, and “5 million yen and over”. The self-rated health status was a dichotomous variable of “good” and “fair/poor”.

### 2.3. Data Analysis

Prior to data analysis, the baseline characteristics of the participants were examined. Furthermore, we examined the differences in baseline characteristics between “maintained” and “declined” by using the Chi-square test or Fisher’s exact test (when the expected score was <5) for categorical variables, and the Mann-Whitney U test for continuous variables in assessing for statistical significance of differences, with *p* < 0.05. We then examined differences in generativity scores based on demographic characteristics of the participants, using the Mann-Whitney U test for binary category variables and the Kruskal-Wallis test for the annual household income variable. Thereafter, binary logistic regression analysis was conducted to examine the relationship between generativity and the decline in TMIG-IC scores over 2 years, controlling for potentially confounding factors (Model 1). We then added the interaction term between generativity and sex (Model 2). The estimates represent the odds ratio (OR) with 95% confidence intervals (CI). The level of significance was set at *p* < 0.05. The data were analyzed using IBM SPSS Statistics 23.

## 3. Results

### 3.1. Participants’ Characteristics and Differences in Baseline Characteristics between “Maintained” and “Declined” Categories

Table 1 illustrates the demographic characteristics of the participants and the differences in baseline characteristics between “maintained” and “declined.” While 639 participants (72.7%) maintained their HLFC over 2 years, 240 participants (27.3%) witnessed a decline. The baseline characteristics were similar between the “maintained” and “declined” categories. The mean baseline TMIG-IC score of the “declined” category was higher (11.89 ± 1.47) than the “maintained” category (11.60 ± 1.68).

### 3.2. Participants’ Generativity

Table 2 illustrates the differences in generativity based on demographic characteristics. The mean generativity score of the “maintained” category was higher (2.42 ± 1.01) than the “declined” category (2.25 ± 1.00), although the difference was not statistically significant. Male participants tended to have a higher generativity score (2.44 ± 1.03) than female participants (2.32 ± 0.99), although the difference was not statistically significant. Age did not show any statistically significant difference in the generativity score. The mean score was higher for participants who were “over high school graduates” (2.50 ± 1.03) than for “high school graduates or under” (2.30 ± 0.98). Participants with “over 5 million yen” (2.59 ± 1.01) reported the highest generativity score among the four categories. Participants who rated their health status as “poor” tended to have higher generativity scores than their counterparts (2.44 ± 0.99, 2.00 ± 1.04, respectively).

### 3.3. Association between Generativity and Change in HLFC over 2 Years

We first conducted Spearman’s rank correlation coefficient to determine multicollinearity among independent variables, and the Pearson correlation coefficients were *r* = −0.16–0.39. Thus, we entered all covariates into the analyses. Table 3 illustrates results of the binary logistic regression analysis. Higher scores on the JGS-R were negatively associated with HLFC decline over 2 years (OR = 0.70, 95%CI = 0.58–0.84), indicating that lower generativity predicted HLFC decline for 2 years. We added the interaction term of generativity with sex in Model 2, and the influence of generativity on HLFC remained significant (OR = 0.69, 95%CI = 0.57–0.83). The interaction term of generativity and sex also reached significance (OR = 1.47, 95%CI = 1.05–2.06), which indicated that the influence of generativity on HLFC decline differed by sex.

To see how the influence of generativity differs by sex, we examined these differences in the mean probability of HLFC decline among participants with higher generativity, as well as among participants with lower generativity, using the Mann-Whitney U test. Participants were categorized as having lower generativity if their generativity score was lower than the mean score (mean = 2.38). There were no differences between the sexes among the participants with lower generativity scores (Mean = 0.29 for women and 0.31 for men). Contrastingly, men had a lower probability of HLFC decline (mean = 0.23) than women (mean = 0.26) among participants with higher generativity (*p* < 0.001).

## 4. Discussion

This study examined the longitudinal association between generativity and HLFC decline over 2 years among older Japanese adults. First, we examined the differences in generativity scores based on demographic characteristics. The results indicated that older adults with higher educational backgrounds and annual household incomes had higher generativity scores than their counterparts. Higher educational attainment and income are associated with participation in social activities [22,23], which may provide them with opportunities to develop generativity. The results also indicated that participants with poorer self-rated health had higher generativity scores than their counterparts. A characteristic of generativity is that individuals can be generative in a variety of life settings, including informal interactions with friends, neighbors, and family members [3,5,6,7,8,9]. Thus, individuals may choose to engage in a variety of generative activities and express generative concerns despite experiencing health decline. It is also assumed that participants with poorer self-rated health may strengthen their generative desire to have a lasting, positive impact on their community and families and friends.

Regarding the longitudinal relationship between generativity and HLFC decline, binary logistic regression analysis showed that a higher score on the JGS-R was negatively associated with a decline in TMIG-IC. This indicates that generativity is effective in preventing HLFC decline over 2 years. Generative activities encompass productive engagements, social participation, and meaningful interactions [6], which are reported to be beneficial for maintaining autonomy and quality of later life [24,25]. Simultaneously, generativity itself motivates older adults to engage in contributive and productive activities and behaviors toward others and their community. Indeed, previous studies have reported that increased generativity is positively associated with volunteering [26]. Thus, higher generativity can produce a positive cycle to maintain or improve older adults’ well-being.

Furthermore, the result of the binary logistic regression analysis, which examined the interaction term of sex, showed that among the participants with higher generativity, men had a lower probability of HLFC decline than women. This implies that generativity is especially effective in protecting the HLFC decline in men with higher generativity. This result contradicts those of previous studies that found generativity to be more predictive of psychological health of older women than older men [8,19]. This result can be understood in relation with traditional gender roles. Women and men emphasize different components of generativity governed by their gender roles, with public contribution (i.e., work and civic activity) and family responsibilities dominating men’s and women’s lives, respectively [27,28]. As Japanese men are expected to be breadwinners and providers for their families [29], they tend to derive meaning of life and identity from the public domain, especially work [28]. Therefore, retirement is often associated with the loss of social roles, opportunities to feel productive, and being a contributing member of the society [30]. Older men with higher generativity may perform satisfactory roles and have opportunities to feel productive and useful in their community. Thus, fulfilling generativity in the public domain may be especially important for men to prevent HLFC decline.

This study’s findings have policy implications for social programs that promote older adults’ health and long-term care prevention. The study results highlighted the importance of developing social programs and activities for older adults so that they can feel productive, contribute to community, and have meaningful interactions with younger generations. Specifically, this may be true for men considering that generativity is especially effective in protecting the HLFC decline in men with higher generativity as observed in this study. Moreover, social activities, generally preferred by men, involve generative engagements that may promote social participation among older men, boosting their productivity and contribution to the society [31]. In the current society, particularly in urban areas, there are limited opportunities for people to engage in intergenerational interactions in their own community; thus, it is important for public health professionals to develop social programs and activities that intentionally foster meaningful intergenerational interactions [32]. Lastly, public health professionals should provide opportunities for older adults with poorer self-rated health to be generative to prevent further HLFC decline.

This study had several limitations. The first corresponds to the generalizability of the results owing to the data collection method. A longitudinal study was conducted in Tokyo metropolitan areas, and the participants were asked to complete questionaries that aimed at examining their attitudes and behaviors toward intergenerational relationships; these participants may have had more interest in intergenerational relationships than the general population. Thus, the study findings may be relevant to only older adults with higher generativity in urban areas. Therefore, future studies should examine the effects of generativity on HLFC in a larger, more representative sample, inclusive of other populations and areas, such as rural areas. Second, this study did not explore processes through which generativity relates to the maintenance of HLFC. A qualitative study may provide us with deeper understanding about these processes, in which generativity or absence of generativity (stagnation) relates to older adults’ actions and behaviors for maintenance or decline in HLFC. Lastly, this study examined the association between HLFC and generativity. However, generativity may have positive associations with other functions, such as cognitive ability, given its nature that involves productive and creative activities. Therefore, future research should examine the association between generativity and other functions, such as cognitive ability.

## 5. Conclusions

This study examined the longitudinal association between generativity and HLFC decline over 2 years among older Japanese adults. The study findings suggest that having higher generativity is effective in preventing HLFC decline over 2 years. Furthermore, generativity is especially effective in protecting the HLFC decline in men with higher generativity. The current study adds to the existing evidence about the protective effect of generativity on the maintenance of HLFC among older adults living in urban areas of Japan. These study results have public health policy implications for social programs that should be developed for older adults where they can capitalize on their skills and experiences, as well as have intergenerational interactions.

## Figures and Tables

**Table 1:** Baseline characteristics of participants and results of univariate analyses between baseline explanatory variables and HLFC maintenance/decline.

	All Participants (*n* = 879)	Maintained (*n* = 639; 72.7%)	Declined (*n* = 240; 27.3%)	*p*-Value *^1^
Sex				0.595
Women	482 (54.8)	354 (55.4)	128 (53.3)	
Men	397 (45.2)	285 (44.6)	112 (46.7)	
Age				0.150
65~74	580 (66.00)	431 (67.40)	149 (62.10)	
75~84	299 (34.00)	208 (32.60)	91 (37.90)	
Have/do not have children				0.430
Have	755 (86.9)	552 (87.5)	203 (85.3)	
Do not have	114 (13.1)	79 (12.5)	35 (14.7)	
Area				0.688
Tama Ward	587 (66.8)	424 (66.4)	163 (67.9)	
Kita Ward	292 (33.2)	215 (33.6)	77 (32.1)	
Highest educational level *^2^				0.586
High school graduate or under	502 (58.30)	370 (58.90)	132 (56.70)	
Over high school graduate	359 (41.70)	258 (41.10)	101 (43.30)	
Annual household income				0.572
<2,000,000 yen	161(19.6)	113 (18.80)	48 (21.70)	
2,000,000 yen–<3,000,000 yen	246 (29.9)	176 (29.20)	70 (31.70)	
3,000,000 yen–<5,000,000 yen	235 (28.60)	176 (29.20)	59 (26.70)	
>5,000,000 yen	181 (22.00)	137 (22.80)	44 (19.90)	
Self-rated health				
Good	735 (85.10)	535 (85.20)	200 (84.70)	0.473
Poor	129 (14.90)	93 (14.80)	36 (15.30)	
Total score of TMIG-IC *^3^	11.68 ± 1.63	11.60 ± 1.68	11.89 ± 1.47	<0.016

*^1^ Chi-square test or Fisher’s exact test (when the expected score was <5) was applied for categorical variables and Mann-Whitney U test for continuous variables (total score of generativity and total score of TMIG-IC). *^2^ “High school graduate or under” includes grade school, junior high school, and high school graduate. “Over high school graduate” includes vocational school, two-years of college, university, or graduate school. *^3^ Total score of TMIG ranges from 0 to 13.

**Table 2:** Total generativity score by participants’ demographic characteristics.

	Generativity Score M ± SD	*p*-Value *^1^
Change in TMIG-IC		
Maintained	2.42 ± 1.01	0.057
Declined	2.25 ± 1.00	
Sex		0.054
Women	2.32 ± 0.99	
Men	2.44 ± 1.03	
Age		0.814
65~74	2.38 ± 0.97	
75~84	2.37 ± 1.08	
Have/do not have children		0.085
Have	2.40 ± 1.00	
Do not have	2.23 ± 1.08	
Area		0.203
Tama Ward	2.35 ± 1.01	
Kita Ward	2.43 ± 1.01	
Highest educational level *^2^		0.009
High school graduate or under	2.30 ± 0.98	
Over high school graduate	2.50 ± 1.03	
Annual household income		0.005
<2,000,000 yen	2.23 ± 0.96	
2,000,000 yen–<3,000,000 yen	2.36 ± 1.02	
3,000,000 yen–<5,000,000 yen	2.37 ± 0.99	
>5,000,000 yen	2.59 ± 1.01	
Self-rated health		
Good	2.00 ± 1.04	0.000
Poor	2.44 ± 0.99	

*^1^ *p*-values were obtained using the Mann-Whitney U tests for binary category variables and the Kruskal-Wallis test for the annual household income variable. *^2^ “High school graduate or under” includes grade school, junior high school, and high school graduate. “Over high school graduate” includes vocational school, two-years of college, university, or graduate school.

**Table 3:** Adjusted odds ratios for predicting decline in the total score of TMIG-IC during the two-year follow-up period.

	Model 1		Model 2	
	OR	95% CI Lower–Upper	OR	95% CI Lower–Upper
Total score of generativity (increment by 1 point)	0.70	0.58–0.84	0.69	0.57–0.83
Sex (ref. Women)				
Men	1.49	1.05–2.11	1.52	1.06–2.16
Age (ref. 65–74 yrs.)				
Over 75 yrs.	1.52	1.07–2.16	1.52	1.07–2.16
Have/do not have children (ref. Have)				
Do not have	1.39	0.86–2.22	1.36	0.85–2.19
Area (ref. Tama Ward)				
Kita Ward	0.94	0.66–1.33	0.94	0.66–1.33
Highest Educational Level (ref. High school graduate or under)				
Over high school graduate	1.12	0.79–1.58	1.15	0.81–1.63
Annual household income (ref. < 2,000,000 yen)				
2,000,000 yen–<3,000,000 yen	0.76	0.48–1.22	0.75	0.47–1.20
3,000,000 yen–< 5,000,000 yen	0.67	0.41–1.09	0.66	0.40–1.08
>5,000,000 yen	0.69	0.41–1.17	0.67	0.40–1.14
Self-rated health (ref. good)				
Poor	1.29	0.80–2.06	1.31	0.82–2.10
Total score of TMIG-IC (increment by 1 point)	1.41	1.22–1.64	1.40	1.21–1.63
Generativity * sex			1.47	1.05–2.06

Note: CI, 95% confidence interval; OR, odds ratio; OR > 1, increased likelihood of decline in TMIG-IC at the follow-up survey. * Dependent variable: maintenance/decline in the total score of TMIG-IC, 0 = the follow-up survey score was unchanged or improved from the baseline survey, 1 = the follow-up survey score decreased by 1 point or more from the baseline survey.

## Data Availability

The data presented in this study are available upon request from the corresponding author.
